# Supplementary material for: Methylation Biomarkers of Lung Cancer Risk: A Systematic Review and Meta-Analysis
Source: Cancers (Basel). 2025 Feb 18;17(4):690. doi: 10.3390/cancers17040690 (PMC11853407; doi:10.3390/cancers17040690)
Supplement: Supplementary file 1 [file cancers-17-00690-s001.zip › Table S1.pdf]

Supplementary Table S1. Search strategy.

**Database**	**Search Details**
**PubMed**	("dna methylation"[MeSH Terms] OR ("dna"[All Fields] AND "methylation"[All Fields]) OR "dna methylation"[All Fields]) AND (("lung neoplasms"[MeSH Terms] OR ("lung"[All Fields] AND "neoplasms"[All Fields]) OR "lung neoplasms"[All Fields] OR ("lung"[All Fields] AND "cancer"[All Fields]) OR "lung cancer"[All Fields]) AND ("risk"[MeSH Terms] OR "risk"[All Fields]))
**Web of Science**	(ALL=(DNA methylation)) AND ALL=(lung cancer risk)
**Scopus**	(ALL (dna AND methylation) AND ALL (lung AND cancer AND risk))
