# Supplementary material for: Methylation Biomarkers of Lung Cancer Risk: A Systematic Review and Meta-Analysis
Source: Cancers (Basel). 2025 Feb 18;17(4):690. doi: 10.3390/cancers17040690 (PMC11853407; doi:10.3390/cancers17040690)
Supplement: Supplementary file 1 [file cancers-17-00690-s001.zip › Table S2.pdf]

Supplementary Table S2: New Castle Ottawa Scale of the studied selected of the Cohort studies.

Cohort Study	Representativeness of the Exposed Cohort	Selection of the Unexposed Cohort	Ascertainment of Exposure	Outcome of Interest not Present at Start of Study	Control for		Assessment of Outcome	Follow-up Long Enough for Outcomes to Occur	Adequacy of Follow up of Cohorts	Total Quality Score
					Important Factor	Additional Factors				
**metDNA**										
Li X, 2022, Germany (66)	1	1	1	0	1	1	1	1	1	8
Hillary RF, 2020 Scotland (194)	1	1	1	0	1	1	1	1	1	8
