# Supplementary material for: Methylation Biomarkers of Lung Cancer Risk: A Systematic Review and Meta-Analysis
Source: Cancers (Basel). 2025 Feb 18;17(4):690. doi: 10.3390/cancers17040690 (PMC11853407; doi:10.3390/cancers17040690)
Supplement: Supplementary file 1 [file cancers-17-00690-s001.zip › Table S3.pdf]

Supplementary Table S3: New Castle Ottawa Scale of the studied selected of the Case-Control studies.

Case-Control Study	Adequate Definition of Cases	Representativeness of Cases	Selection of Controls	Definition of Controls	Comparability		Ascertainment Exposure	Same Method of Ascertainment for Cases and Controls	Non-Response Rate	Total Quality Score
					Important Factor	Additional Factors				
**metDNA**										
Michaud DS, 2023, USA (27)	1	1	1	1	0	1	1	1	1	8
Dugue PA, 2023 Australia, MCCS (45)	1	1	1	1	0	1	1	1	1	8
Dugue AP, 2021 Australia, MCCS (160)	0	1	1	1	1	1	1	1	1	8
Zhao N, 2021 Washington County, USA (161)	1	1	1	1	1	1	1	1	1	9

Dugue PA, 2020 Australia, MCCS (129)	0	1	1	1	1	1	1	1	1	8
Yu H, 2020 Saarland, Germany (219)	1	1	1	1	1	1	1	1	1	9
Gagliardi A. 2020, Italy, Australia, Norway (234)	1	1	1	1	1	1	1	1	1	9
Dugue AP 2018, Melbourne Australia (293)	0	1	1	1	1	1	1	1	1	8
Zhang Y 2016 Saarland, Germany (378)	0	1	1	1	1	1	1	1	1	8
Levine ME, 2015, USA (413)	0	1	1	0	1	1	1	1	1	7
