# Supplementary material for: Methylation Biomarkers of Lung Cancer Risk: A Systematic Review and Meta-Analysis
Source: Cancers (Basel). 2025 Feb 18;17(4):690. doi: 10.3390/cancers17040690 (PMC11853407; doi:10.3390/cancers17040690)
Supplement: Supplementary file 1 [file cancers-17-00690-s001.zip › Table S4.pdf]

Table S4. Reasons of the excluded studies.

Reason of exclusion	Motivation
Single gene methylation	Methylation is typically studied at the single gene level. However, regression models for methylation generally consider whole genome activity, which is why we did not focus exclusively on single gene methylation.
Animal model	Our systematic review and meta-analysis are focused on human studies, and animal models were not included in our systematic review and meta-analysis.
Promoter methylation	Methylation as an epigenetic mechanism is not limited to the promoter region. It can also occur in other regions of the gene, which is why we did not limit our systematic review and meta-analysis to only promoter methylation.
Mendelian Randomization	The Newcastle-Ottawa Scale (NOS) cannot be applied to randomized studies like Mendelian randomization, so these articles were excluded from this systematic review and meta-analysis.
