# Supplementary material for: Methylation Biomarkers of Lung Cancer Risk: A Systematic Review and Meta-Analysis
Source: Cancers (Basel). 2025 Feb 18;17(4):690. doi: 10.3390/cancers17040690 (PMC11853407; doi:10.3390/cancers17040690)
Supplement: Supplementary file 1 [file cancers-17-00690-s001.zip › Table S5.pdf]

Table S5. Definition of DNA methylation measure.

Type of DNA methylation measure	Definition
**AA_Grim**	**Age Acceleration Grim**: is an epigenetic clock designed to predict lifespan and health span. DNAm GrimAge stands out among existing epigenetic clocks in terms of its predictive ability for time-to-death, time-to-coronary heart disease, time-to-cancer, its strong relationship with computed tomography data for fatty liver/excess visceral fat, and age-at-menopause [1].
**AA_Hannum**	**Age Acceleration Hannum**: is an epigenetic clock based on the methylation markers identified by Hannum et al. in 2013. This clock uses specific DNA methylation patterns to estimate biological age as an indicator of age-related health decline [2].
**AA_Horvath**	**Age Acceleration Horvath**: is derived from the Horvath epigenetic clock. This novel epigenetic clock can be used to address a host of questions in developmental biology, cancer, and aging research [3].
**AA_Pheno**	**Age Acceleration Pheno**: is an epigenetic clock that predicts aging by using DNA methylation markers correlated with a set of clinical biomarkers [4].
**IEAA_Hannum**	**Intrinsic Epigenetic Age Acceleration Hannum**: is a biological age measurement defined as the residual resulting from regressing epigenetic age on chronological age and measures of blood cell counts [5].
**IEAA_Horvath**	**Intrinsic Epigenetic Age Acceleration Horvath**: is a biological age measurement focused on intrinsic aging processes, derived from the Horvath clock. It measures cellular age acceleration independently of blood cell proportions, which are known to change with age [6,7].
**IEAA_Pheno**	**Intrinsic Epigenetic Age Acceleration Pheno**: is a biological age measurement derived from the PhenoAge clock, focusing on the "intrinsic" aspects of aging. It isolates the biological aging process from external influences like lifestyle or temporary health conditions [4].

[1] A. T. Lu *et al.*, "DNA methylation GrimAge strongly predicts lifespan and healthspan," *Aging (Albany NY)*, vol. 11, no. 2, p. 303, Jan. 2019, doi: 10.18632/AGING.101684.

[2] G. Hannum *et al.*, "Genome-wide Methylation Profiles Reveal Quantitative Views of Human Aging Rates," *Mol Cell*, vol. 49, no. 2, pp. 359–367, Jan. 2013, doi: 10.1016/J.MOLCEL.2012.10.016.

[3] S. Horvath, "DNA methylation age of human tissues and cell types," *Genome Biol*, vol. 14, no. 10, pp. 1–20, Oct. 2013, doi: 10.1186/GB-2013-14-10-R115/COMMENTS.

[4] M. E. Levine *et al.*, "An epigenetic biomarker of aging for lifespan and healthspan," *Aging (Albany NY)*, vol. 10, no. 4, p. 573, Apr. 2018, doi: 10.18632/AGING.101414.

[5] B. H. Chen *et al.*, "DNA methylation-based measures of biological age: meta-analysis predicting time to death," *Aging (Albany NY)*, vol. 8, no. 9, p. 1844, 2016, doi: 10.18632/AGING.101020.

[6] S. Horvath *et al.*, "Obesity accelerates epigenetic aging of human liver," *Proc Natl Acad Sci U S A*, vol. 111, no. 43, pp. 15538–15543, Oct. 2014, doi: 10.1073/PNAS.1412759111/SUPPL_FILE/PNAS.1412759111.SD01.XLSX.

[7] J. A. Smith *et al.*, “Intrinsic and extrinsic epigenetic age acceleration are associated with hypertensive target organ damage in older African Americans,” *BMC Med Genomics*, vol. 12, no. 1, pp. 1–12, Oct. 2019, doi: 10.1186/S12920-019-0585-5/TABLES/3.
